# Development and validation of an enteral feeding interruption management scale for ICU medical staff: A knowledge‐, attitude‐ and practice‐based approach

**DOI:** 10.1111/nicc.70024

**Published:** 2025-04-02

**Authors:** Yuanyuan Mi, Fei Tian, Lifei Wang, Chenglin Xiang, Liang Sun

**Affiliations:** ^1^ Department of Intensive Care Medicine, Union Hospital, Tongji Medical College Huazhong University of Science and Technology Wuhan People's Republic of China; ^2^ The First College of Clinical Medical Science, China Three Gorges University Teaching and Research Section of Clinical Nursing Yichang People's Republic of China; ^3^ Faculty of Nursing Mahidol University Bangkok Thailand; ^4^ Department of Intensive Care Medicine, The Sixth Hospital of Wuhan Affiliated Hospital of Jianghan University Wuhan People's Republic of China

**Keywords:** enteral feeding interruption, enteral nutrition, intensive care, reliability, validity

## Abstract

**Background:**

Enteral feeding interruption (EFI) is a frequent issue in ICU settings, affecting nutritional adequacy and delaying recovery in critically ill patients. While tools exist to assess patients' nutritional status, no instrument evaluates ICU staff's knowledge, attitude and practice (KAP) in EFI management.

**Aim:**

To develop a reliable and valid EFI management scale for ICU medical staff based on the KAP model.

**Study Design:**

This instrument development study followed the STROBE guidelines, utilizing a cross‐sectional, multi‐centre approach in Wuhan. A convenience sample of 400 ICU staff from eight tertiary A hospitals and two tertiary B hospitals was included between May 2021 and March 2022. A preliminary scale was constructed through literature review, interviews and expert consultations. The sample was used to assess the scale's reliability and validity.

**Results:**

The final EFI management scale comprised 41 items across three dimensions, with cumulative variance contributions of 70.341%, 70.437% and 66.550%. Cronbach's α ranged from 0.919 to 0.947, with test–retest reliability between 0.488 and 0.836. The total scale had a Cronbach's α of 0.953 and test–retest reliability of 0.977. Content validity indices (I‐CVI) ranged from 0.800 to 1.000, and the scale‐level CVI was 0.975.

**Conclusions:**

The EFI Management KAP Scale is a valid, reliable tool for assessing ICU medical staff's management of EFI.

**Relevance to Clinical Practice:**

The EFI Management KAP Scale addresses a critical gap in the standardized evaluation of critical care nurses' knowledge, attitudes and practices regarding enteral feeding interruptions. By providing a validated tool, this scale enables the identification of specific barriers and facilitators to optimal enteral nutrition delivery in critically ill patients. Its application can guide targeted educational interventions, inform policy adjustments and enhance multidisciplinary collaboration in the ICU. Ultimately, this scale supports improved enteral nutrition management, reducing complications associated with feeding interruptions and contributing to better patient outcomes in critical care settings.

**What is known about the topic**

- Enteral feeding interruptions are common in ICU settings and are associated with adverse patient outcomes, including malnutrition and increased morbidity.
- Effective management of feeding interruptions requires ICU medical staff to have adequate knowledge, positive attitudes and appropriate practices.
- There is no validated tool to comprehensively assess ICU staff's knowledge, attitudes and practices related to managing enteral feeding interruptions.

**What this paper adds**

- This study presents the development and validation of a novel “Enteral Feeding Interruption Management Scale,” specifically designed for ICU medical staff.
- The scale provides a standardized method to evaluate and identify gaps in knowledge, attitudes and practices, thereby facilitating targeted interventions.
- Its application can inform clinical education, enhance evidence‐based feeding protocols and ultimately improve nutritional outcomes for critically ill patients.

## 1 INTRODUCTION

Enteral nutrition (EN) is a method of delivering essential nutrients via a feeding tube to meet human metabolic requirements.^1^, ^2^ It is a cornerstone of nutritional support therapy for critically ill patients, leveraging gastrointestinal functionality to modulate the microbiota, preserve intestinal mucosal barriers, reduce infection rates, enhance immune responses and promote gastrointestinal motility.^3^, ^4^ Recognizing its critical importance, the American Society for Parenteral and Enteral Nutrition (ASPEN) recommended EN as the primary nutritional support for critically ill adults in their 2016 guidelines.^5^ Early initiation of enteral nutrition (EN) not only supports nutritional status but also maintains intestinal mucosal integrity, aiding recovery. Similarly, the 2019 ESPEN guidelines advocate for the continuous provision of EN to avoid interruptions and optimize patient outcomes.^6^ Despite these recommendations, EN interruptions remain a significant challenge in intensive care unit (ICU) settings. Acute gastrointestinal injury (AGI), often triggered by severe trauma, hemodynamic instability, or physiological stress, is a frequent cause of these interruptions.^7^, ^8^ Reports indicate that EN interruptions affect 54%–84% of ICU patients and occur during 38%–100% of their ICU stay,^8^ reducing energy intake and impacting outcomes. Lee et al. reported energy deficits of 1780.23 kcal and protein losses of 100.58 g due to these interruptions.^7^ Such deficits can impair immune function, delay wound healing, increase infection risk, prolong ICU stays and elevate mortality rates.

ICU physicians and nurses play crucial roles in formulating EN plans, assessing nutritional needs, monitoring complications and evaluating outcomes.^9^, ^10^, ^11^ However, the knowledge, attitudes and practices (KAP) of health care professionals regarding EN interruptions remain underexplored. KAP assessment tools are widely used in health care research to evaluate and enhance professional competencies and behaviours by identifying gaps and targeting interventions. For example, KAP frameworks have been instrumental in improving adherence to infection control measures, occupational health and safety, and enhancing waste management practices.^12^, ^13^, ^14^ By understanding health care workers' knowledge and perceptions, targeted educational and behavioural interventions can be designed to improve clinical practices and patient outcomes.

Currently, there is a global scarcity of validated tools specifically addressing KAP related to EN interruptions in ICU settings. This gap limits the ability to systematically assess and address the factors contributing to EN interruptions. Developing an ICU‐specific EN interruption KAP assessment tool would facilitate the identification of knowledge deficits, attitudinal barriers and suboptimal practices among health care professionals. Furthermore, such a tool could guide the development of targeted training programmes, enhance adherence to evidence‐based EN protocols and ultimately reduce the frequency and impact of EN interruptions. Addressing this unmet need is essential for improving the nutritional management and clinical outcomes of critically ill patients.

### 1.1 Aims and research questions

This study aims to develop an ICU‐specific Enteral Nutrition (EN) interruption knowledge, attitudes and practices (KAP) scale, focusing on ICU health care professionals' perspectives. The research addressed the following questions:

- Is the scale a valid tool for assessing ICU staff's KAP regarding enteral feeding interruption management?
- Is the scale a reliable measure for evaluating ICU staff's KAP in this context?

## 2 METHOD

### 2.1 Design, setting and sample

A cross‐sectional, descriptive multi‐centre study was conducted in Wuhan to evaluate the validity and reliability of the EFI Management KAP Scale for ICU medical staff. The study adhered to the STROBE guidelines, and a convenience sampling method was used. As recommended in the literature, the sample size in clinimetric studies is usually set considering at least 5–10 subjects for each item^15^, ^16^; the final 49‐item scale required 245–490 participants. To account for potential invalid responses, the sample size was increased by 15%. A total of 400 ICU health care workers from 8 tertiary‐level A and 2 tertiary‐level B hospitals in Wuhan were recruited between May 2021 and March 2022. Inclusion criteria: (1) age >18 years; (2) having a health professional qualification; (3) being employed in the ICU; (4) having the ability to comprehend scale items accurately; (5) informed consent and willingness to participate. Exclusion criteria included ICU vacationers or staff involved in rotations, training, or further medical education. In the qualitative interviews part, purposive sampling procedures were used, and participants were deliberately invited due to their unique insights related to their experience with EN^17^; sampling was considered complete when no new significant themes emerged during the interviews.

### 2.2 Scale development

#### 2.2.1 Establishment of the research team

The research team comprised an ICU clinical administrator with a deputy senior title and master's supervisor qualification, a deputy chief physician with a senior title and PhD, two clinical researchers with master's degrees and three ICU clinical staff members with bachelor's degrees. The team's responsibilities included creating the initial item pool through literature reviews and interviews, selecting experts, distributing and collecting expert surveys, refining scale items, and performing item analysis and reliability testing to finalize the KAP scale.

#### 2.2.2 Item generation

Based on the knowledge, attitude and practice (KAP) model, a systematic literature search was conducted in databases including CNKI, Wanfang, China Biomedical Literature (CBM), PubMed, EmBase, Web of Science and CINAHL. Search terms such as “critically ill patients,” “enteral nutrition,” and “feeding interruption” were used. Additionally, we reviewed relevant materials from professional organizations such as the Chinese Society of Enteral and Parenteral Nutrition, the Chinese Society of Critical Care Medicine, and international bodies like the European and American Societies for Clinical Nutrition and Critical Care.

To align the scale with the clinical and cultural context of critical care in China, in‐depth interviews were conducted in June 2021, including four ICU clinicians, three nutritional support nurses and five ICU nursing team leaders from a tertiary hospital in Wuhan. Topics included the significance of enteral nutrition, perceptions of feeding interruptions, causes, strategies for managing interruptions and their impact on patients. These interviews were recorded using a digital audio recorder to ensure high‐quality data capture. The recordings were transcribed verbatim, maintaining accuracy and capturing nuances in language and expression. Transcriptions were then subjected to thematic analysis, a method well suited for identifying and interpreting patterns within qualitative data.^18^ The analysis process began with multiple readings of the transcripts to achieve data familiarization. Initial codes were generated to identify significant statements and concepts related to the interview topics. Both inductive and deductive coding approaches were employed: inductive coding allowed for the emergence of themes directly from the data, while deductive coding was guided by the Knowledge‐Attitude‐Practice (KAP) framework. These codes were organized into overarching themes and subthemes that captured participants' insights into EN interruptions. The identified themes were directly used for item generation. Item generation refers to the process of creating specific questions or statements (items) that reflect the constructs being measured. In this study, the process aimed to develop items that assess ICU health care professionals' knowledge, attitudes and practices regarding EN interruptions. Each item was crafted to be clear, concise and contextually relevant, ensuring that it accurately reflected the qualitative findings and aligned with the theoretical framework. This methodology is supported by best practices in scale development, where qualitative data provides a foundation for generating items that are both comprehensive and representative of the construct of interest.^19^ By integrating participants' experiences and professional insights, the resulting scale ensures contextual relevance and practical applicability in critical care settings.

Referencing key publications such as the 2016 ASPEN guideline,^5^ the 2018 CSPEN expert consensus^20^ and the 2019 ESPEN guideline,^6^ our research team previously developed an expert consensus on common complications of enteral nutrition in critically ill patients,^21^ which was also included, and a preliminary pool of pre‐test scale entries was formulated. This pool comprised three dimensions: knowledge (16 entries), attitudes (10 entries), and practices (24 entries), totalling 50 entries. Following group discussion and analysis, combined with interview outcomes, this initial scale pool was established.

#### 2.2.3 Expert consultations

Expert consultations were conducted to assess the content validity of the ICU Enteral Nutrition Feeding Disruption scale. Two rounds of consultations were distributed via WeChat QR codes. Experts rated the importance of scale items using a 5‐point Likert scale, with a column for suggested revisions. Items scoring ≤3.50 or with a coefficient of variation ≥0.25 were marked for potential revision or deletion. Selection criteria required experts to have a Bachelor's degree or higher, an intermediate or senior title, at least 10 years of experience (or 5 years with a master's degree), and expertise in ICU or clinical nutrition. Opinions from the first round were consolidated within 3 weeks, followed by a second round of scale refinement. The consultation concluded with 20 experts from 15 tertiary hospitals across multiple provinces, with diverse expertise in clinical management, intensive care, nursing, medicine, surgery and emergency care.

#### 2.2.4 Pilot survey

In July 2021, 50 health care workers were selected from a tertiary hospital in Wuhan to pre‐test the scale. The aim was to assess the linguistic accuracy, semantic clarity and logical arrangement of the scale entries. After completing the pre‐test of the scale, a cross‐sectional, descriptive multi‐centre study was conducted in Wuhan to evaluate the validity and reliability of the EFI Management KAP Scale for ICU medical staff.

### 2.3 Data collection

#### 2.3.1 Data collection tool

Data collection tool consists of two sections: the first section captures participants' general information, including gender, age, education, professional title, ICU experience, ICU type, membership in nutritional support groups, enteral feeding interruption training experience and knowledge level of enteral nutrition. The second section is the ICU Medical Staff EFI knowledge, attitude and practice (KAP) scale, consisting of 49 items across three dimensions: knowledge (16 items), attitude (10 items) and practice (23 items). Each item is rated on a five‐point Likert scale. Knowledge items range from 1 (completely uninformed) to 5 (completely understood), attitude items range from 1 (completely disagree) to 5 (completely agree), and practice items range from 1 (never) to 5 (always).

#### 2.3.2 Data collection process

The Scale Star platform was used for distributing and collecting data. ICU directors and head nurses from 10 Wuhan hospitals were informed about the study's objectives, content and criteria. After obtaining consent, the survey was shared via a QR code. Standardized instructions clarified the study's purpose, completion methods and guidelines. To ensure data authenticity and prevent duplicate entries, each mobile device was restricted to a single response. The survey remained open for 2 weeks, after which access was closed automatically. Data were monitored and validated for completeness and accuracy. To assess test–retest reliability, 60 health care professionals retook the survey 30 days later.

### 2.4 Statistical analysis

Data from the scale were validated by two individuals and analysed using SPSS 22.0. Exploratory factor analysis (EFA) was performed to provide evidence for the construct validity of the scale. Before EFA, the Kaiser–Meyer–Olkin (KMO) measure of sampling adequacy and Bartlett's test of sphericity were applied to determine whether the data were suitable for principal components analysis. For the qualitative data, thematic analysis was employed to identify and interpret patterns. The interviewer listened to the recordings, read the transcripts and conducted line‐by‐line coding after each interview. The notes were organized into categories of knowledge, attitude and practice using Microsoft Excel. These categories were subsequently discussed until a consensus was reached.

#### 2.4.1 Item analysis

Discriminant analysis, question‐total correlation and principal component analysis were applied to evaluate scale item adequacy and consistency.^22^ Discriminant analysis: The sample was divided into two groups (top and bottom 27% based on total scores). An independent t‐test was performed, and items with *p* < .05 and a decision value (CR) <3.000 were flagged for deletion. Total Correlation Coefficient: Items with Pearson correlation coefficients <0.4 or corrected item‐total correlation (CITC) <0.4 were considered weakly associated and flagged for deletion. Principal Component Analysis: Items with commonality <0.40 were considered for deletion if they were poorly aligned with the scale's common factor.

#### 2.4.2 Validity assessment

Construct Validity: Principal component analysis (PCA) and maximum orthogonal rotation (MORV) were used in exploratory factor analysis (EFA) to define scale structure. Factors with eigenvalues >1.0, contributing to cumulative variance >60%, with at least three items and loadings >0.5 were retained. Content Validity: Experts rated item importance, with a content validity index >0.7 for individual items and >0.9 for the overall scale considered acceptable.

#### 2.4.3 Reliability assessment

Internal Consistency: Cronbach's α >0.700 indicated acceptable reliability, with 0.800–0.900 indicating strong consistency.^16^ Test–Retest Reliability: Sixty respondents were retested after 30 days. A reliability coefficient >0.6 was considered acceptable, with higher values indicating better stability.^23^

### 2.5 Ethical and research approvals

This study was approved by the Ethics Committee of Union Hospital, Tongji Medical College, Huazhong University of Science and Technology (number: UHCT22094, date: 24, 02, 2022).

## 3 RESULTS

Two experts were involved to assess the content validity of the tool, and 400 were used to evaluate the tool.

### 3.1 Results of expert consultation

Twenty clinical experts in critical care, nursing, internal medicine, surgery, emergency care, and clinical nutrition (6 males, 14 females; ages 31–58, mean 43.65 ± 7.55) participated in this study. Eight held bachelor's degrees, 10 had master's degrees and two had PhDs. Their titles ranged from intermediate to senior, with 6–39 years of experience (mean: 21.85 ± 9.77). All 20 questionnaires were returned across two consultation rounds, yielding a 100% response and validity rate. Authority coefficients were 0.92 and 0.93, indicating high expertise. Kendall's harmony coefficient improved from 0.205 in the first round to 0.613 in the second, demonstrating increased consensus.

Experts assessed language, clarity, and item sequence. Importance scores ranged from 4.00 to 5.00, with variation coefficients of 0.00–0.11. Two entries (A5, B15) exceeded a variation coefficient of 0.25, and two others (B14, B21) had differing perspectives. Three entries required sequencing adjustments (K3, K5, K12). After revisions, 1 entry was deleted, 17 were modified and 2 were merged. The final pre‐survey scale contained 49 entries: 16 for knowledge, 10 for attitude and 23 for practice.

### 3.2 Results of pilot survey

The scale's completion time for ICU medical staff ranged from 5 to 12 min, allowing adequate comprehension of each entry. During the pretest, two scales had omitted responses. To address this, a “prevent omission” feature was added to the survey platform, ensuring all entries were completed before submission in the official survey. This adjustment improved data quality and minimized incomplete responses.

### 3.3 Demographic characteristics of the participants

Of the 400 participants, 382 submitted valid questionnaires, yielding a 95.50% valid response rate. The cohort included 112 males and 270 females, aged 21–56 years (mean: 31.77 ± 6.99). It comprised 90 doctors and 292 nurses, with 230 holding junior titles, 105 intermediate titles and 47 senior titles. Additionally, 53.66% (205) had over 5 years of ICU experience, and 349 participants held at least a bachelor's degree. Most participants (81.68%) worked in comprehensive ICUs, with 26.44% involved in enteral nutrition groups. The majority (86.13%) were from tertiary hospitals. The main source of knowledge on enteral nutrition interruptions was hospital/departmental lectures (74.08%), while only 9.69% relied on academic journals. Although 74.61% (285/382) had some knowledge of the topic, it was not comprehensive.

### 3.4 Item analysis results

In Table 1, it revealed decisive values of the scale entries from 8.711 to 18.596, *p* < .001, indicating reasonable scale design with good differentiation. Total correlation coefficients analysis identified entry B15 with a coefficient of 0.375 <0.40, leading to its deletion. Following this deletion, the remaining entries showed correlation coefficients with the total table ranging from 0.429 to 0.693, indicating acceptable item discrimination and internal consistency of the scale.

**TABLE 1:** Results on the measurement model of the scale.

Factor	Item	Discrimination analysis		Total correlation	Factor loadings	Communality
		*t* Values	*p* Values			
Factor 1	K1. An interruption in enteral nutrition feeding occurs when the infusion lasts for 1 h or more without continuous feeding.	9.813	***	0.456	0.831	0.696
	K2. Interruption of enteral nutrition feeding is defined as failure to receive the expected nutrients within 30 min, particularly in intermittent infusion occurring three times a day for 30 min each.	8.991	***	0.429	0.863	0.744
	K3. Post‐enternal nutrition interruptions may impact a patient's energy intake, increasing the risk of nutritional deficiencies.	14.954	***	0.606	0.680	0.650
	K4. Interruptions in enteral nutrition feeding correlate positively with patient severity, hospitalization costs and targeted caloric intake.	15.536	***	0.605	0.751	0.728
Factor 2	K6. Factors such as hemodynamic instability, high intra‐abdominal pressure and digestive‐related complications (intestinal obstruction, anastomotic leakage, celiac disease) significantly contribute to enteral nutrition feeding interruptions.	15.475	***	0.642	0.703	0.682
	K8. Medical and nursing procedures like general anaesthesia, radiology, endotracheal fibre, airway establishment/replacement, position changes and suctioning often cause disruptions in enteral nutritional feeding.	16.288	***	0.660	0.744	0.702
	K9. Challenges such as tube placement difficulty, occlusion, displacement and dislodgement of nutritional infusion tubes are common causes of feeding interruptions in critically ill patients.	16.856	***	0.673	0.725	0.692
	K10. Early initiation of enteral nutrition within 24 to 48 h of admission is recommended for critically ill patients with well‐functioning gastrointestinal tracts and stable hemodynamics.	13.003	***	0.612	0.862	0.762
	K11. Monitoring the gastric remnant is crucial before each feeding for patients receiving enteral nutrition through fractionated push and intermittent gravity drip. For continuous nutrition pump infusion, monitoring should occur at least every 4 h.	12.762	***	0.608	0.817	0.695
	K12. Sedative and analgesic medications may delay gastric emptying. Regular assessment of pain levels and sedation depth is essential, minimizing drug use as per the treatment plan and patient's preferences.	17.743	***	0.675	0.786	0.719
	K13. Pro‐gastrointestinal dynamic drugs may alleviate gastrointestinal intolerance symptoms in critically ill patients.	17.817	***	0.693	0.842	0.780
	K14. For critically ill patients with elevated intra‐abdominal pressure (IAP >12 mmHg), routine monitoring of intra‐abdominal pressure is necessary. Adjusting the infusion rate and volume of enteral nutrition based on pressure levels is recommended.	18.596	***	0.685	0.617	0.599
	K15. Keeping the head of the bed elevated between 30° to 45° during enteral nutrition in critically ill patients is advisable unless contraindicated.	11.821	***	0.591	0.819	0.671
	K16. Resuming enteral nutritional feeding promptly after completing medical procedures, consultations, and other related examinations is recommended.	15.390	***	0.645	0.851	0.728
Factor 3	A1. It is crucial for ICU medical staff to possess knowledge about enteral nutrition feeding interruptions.	9.504	***	0.571	0.800	0.640
	A2. I believe that the hospital (or department) should conduct formal training on knowledge of enteral nutrition tolerance.	11.231	***	0.576	0.815	0.665
	A3. Enhanced knowledge regarding enteral nutrition feeding interruptions significantly aids in my clinical work.	10.908	***	0.599	0.830	0.690
	A4. Conducting nutritional assessments for all admitted ICU patients is regarded as an important practice.	10.579	***	0.592	0.845	0.714
	A5. Health care involvement is necessary for assessing the nutritional status of ICU patients.	8.711	***	0.575	0.888	0.789
	A6. The careful selection of appropriate enteral nutrition preparations/formulas plays a pivotal role in preventing interruptions in enteral nutritional feeding.	10.226	***	0.608	0.912	0.833
	A7. The proper establishment and selection of the infusion route for enteral nutrition are deemed important.	10.655	***	0.607	0.900	0.810
	A8. The management of enteral nutrition positioning holds significant importance.	11.258	***	0.622	0.849	0.721
	A9. Developing a standardized program for enteral nutrition management to prevent and address feeding interruptions is important	11.397	***	0.637	0.880	0.775
	A10. I believe that prioritizing the prevention of interruptions in enteral nutritional feeding outweighs treatment.	10.331	***	0.501	0.639	0.408
Factor 4	B1. I will proactively study relevant knowledge about interruptions in enteral nutrition feeding.	16.333	***	0.630	0.818	0.716
	B2. I will actively communicate with patients or their families about the importance of enteral nutrition, informing them about the risks of interruptions in enteral nutrition feeding.	14.566	***	0.592	0.855	0.769
	B3. Upon admitting ICU patients, I will promptly assess the patient's nutritional status and communicate with the attending physician.	15.653	***	0.643	0.818	0.751
	B4. Before implementing enteral nutrition, I will communicate with the doctor to select and establish the correct enteral nutrition feeding route.	16.816	***	0.670	0.825	0.783
Factor 5	B5. During enteral nutrition implementation, I will strictly adhere to hand hygiene.	11.982	***	0.632	0.700	0.627
	B7. Unless medically contraindicated, I will elevate the head of the bed by 30° to 45° for patients receiving enteral nutrition support.	9.771	***	0.601	0.812	0.707
	B8. During enteral nutrition feeding, I will monitor symptoms of feeding intolerance in patients (such as nausea, vomiting, reflux/aspiration, abdominal distension), and promptly report to the physician.	11.609	***	0.644	0.793	0.731
	B10. I will cease enteral nutrition when patients undergo emergency airway establishment/replacement procedures, such as endotracheal intubation or tracheostomy.	10.071	***	0.566	0.755	0.627
	B11. I will stop enteral nutrition when patients undergo bedside X‐ray examinations or endotracheal fibroscopy.	9.528	***	0.584	0.733	0.632
	B12. In cases where a patient's condition deteriorates requiring immediate surgery or is anticipated to undergo general anaesthesia surgery within 4–8 h, I will halt enteral nutrition.	9.676	***	0.603	0.778	0.698
	B18. In critically ill patients with upper gastrointestinal bleeding or intestinal ischemia, I will discontinue enteral nutrition.	10.169	***	0.598	0.686	0.591
Factor 6	B14. For patients with feeding intolerance, I will investigate and analyse the causes and consult with the physician to jointly decide whether to discontinue enteral nutrition.	15.381	***	0.651	0.553	0.603
	B16. I will halt enteral nutrition for patients in shock with non‐correctable conditions, requiring a gradual increase in vasopressor doses to maintain hemodynamic stability (MAP <50 mmHg).	13.351	***	0.551	0.823	0.742
	B17. For patients experiencing uncontrollable life‐threatening hypoxemia, hypercapnia, or acidosis, I will discontinue enteral nutrition.	12.392	***	0.562	0.798	0.702
	B19. For patients with increased bladder pressure (IAP >20 mmHg), I will discontinue enteral nutrition.	12.406	***	0.523	0.731	0.604
	B21. During enteral nutrition support, if gastric residual volume is monitored twice and exceeds 250 mL, I will remind the physician about the potential use of prokinetic agents.	13.62	***	0.642	0.519	0.535
	B22. For patients intolerant to gastric feeding and ineffective use of prokinetic agents, or those deemed to have a high risk of aspiration, I will communicate with the physician to establish post‐pyloric feeding routes (inserting a “bullet head” nasointestinal tube for the patient).	13.254	***	0.588	0.528	0.495

*** *p* < .001.

The mean values and Cronbach's alpha scores for individual items are given in Table 2. Based on the findings, all items appear to contribute adequately to the reliability of the scale, with no single item negatively impacting overall consistency.

**TABLE 2:** Mean values and Cronbach's alpha scores for individual items.

Factor	Item	Mean	SD	Cronbach's alpha scores
Factor 1	K1. An interruption in enteral nutrition feeding occurs when the infusion lasts for 1 h or more without continuous feeding.	2.843	1.002	0.936
	K2. Interruption of enteral nutrition feeding is defined as failure to receive the expected nutrients within 30 min, particularly in intermittent infusion occurring three times a day for 30 min each.	3.073	1.082	0.939
	K3. Post‐enternal nutrition interruptions may impact a patient's energy intake, increasing the risk of nutritional deficiencies.	2.327	0.994	0.929
	K4. Interruptions in enteral nutrition feeding correlate positively with patient severity, hospitalization costs and targeted caloric intake.	2.356	0.971	0.929
Factor 2	K6. Factors such as hemodynamic instability, high intra‐abdominal pressure and digestion‐related complications (intestinal obstruction, anastomotic leakage, celiac disease) significantly contribute to enteral nutrition feeding interruptions.	2.065	0.904	0.927
	K8. Medical and nursing procedures like general anaesthesia, radiology, endotracheal fibre, airway establishment/replacement, position changes and suctioning often cause disruptions in enteral nutritional feeding.	2.052	0.868	0.926
	K9. Challenges such as tube placement difficulty, occlusion, displacement and dislodgement of nutritional infusion tubes are common causes of feeding interruptions in critically ill patients.	2.058	0.864	0.927
	K10. Early initiation of enteral nutrition within 24 to 48 h of admission is recommended for critically ill patients with well‐functioning gastrointestinal tracts and stable hemodynamics.	1.715	0.854	0.928
	K11. Monitoring the gastric remnant is crucial before each feeding for patients receiving enteral nutrition through fractionated push and intermittent gravity drip. For continuous nutrition pump infusion, monitoring should occur at least every 4 h.	1.846	0.866	0.929
	K12. Sedative and analgesic medications may delay gastric emptying. Regular assessment of pain levels and sedation depth is essential, minimizing drug use as per the treatment plan and patient's preferences.	1.945	0.860	0.926
	K13. Pro‐gastrointestinal dynamic drugs may alleviate gastrointestinal intolerance symptoms in critically ill patients.	1.914	0.845	0.926
	K14. For critically ill patients with elevated intra‐abdominal pressure (IAP >12 mmHg), routine monitoring of intra‐abdominal pressure is necessary. Adjusting the infusion rate and volume of enteral nutrition based on pressure levels is recommended.	2.175	0.941	0.928
	K15. Keeping the head of the bed elevated between 30° to 45° during enteral nutrition in critically ill patients is advisable unless contraindicated.	1.521	0.762	0.932
	K16. Resuming enteral nutritional feeding promptly after completing medical procedures, consultations and other related examinations is recommended.	1.628	0.748	0.930
Factor 3	A1. It is crucial for ICU medical staff to possess knowledge about enteral nutrition feeding interruptions.	1.301	0.557	0.943
	A2. I believe that the hospital (or department) should conduct formal training on knowledge of enteral nutrition tolerance.	1.291	0.524	0.942
	A3. Enhanced knowledge regarding enteral nutrition feeding interruptions significantly aids in my clinical work.	1.312	0.517	0.941
	A4. Conducting nutritional assessments for all admitted ICU patients is regarded as an important practice.	1.275	0.513	0.941
	A5. Health care involvement is necessary for assessing the nutritional status of ICU patients.	1.202	0.439	0.940
	A6. The careful selection of appropriate enteral nutrition preparations/formulas plays a pivotal role in preventing interruptions in enteral nutritional feeding.	1.246	0.472	0.938
	A7. The proper establishment and selection of the infusion route for enteral nutrition are deemed important.	1.243	0.454	0.939
	A8. The management of enteral nutrition positioning holds significant importance.	1.272	0.501	0.941
	A9. Developing a standardized program for enteral nutrition management to prevent and address feeding interruptions is important	1.259	0.473	0.939
	A10. I believe that prioritizing the prevention of interruptions in enteral nutritional feeding outweighs treatment.	1.416	0.654	0.954
Factor 4	B1. I will proactively study relevant knowledge about interruptions in enteral nutrition feeding.	2.325	0.980	0.916
	B2. I will actively communicate with patients or their families about the importance of enteral nutrition, informing them about the risks of interruptions in enteral nutrition feeding.	2.351	1.046	0.916
	B3. Upon admitting ICU patients, I will promptly assess the patient's nutritional status and communicate with the attending physician.	2.016	0.925	0.914
	B4. Before implementing enteral nutrition, I will communicate with the doctor to select and establish the correct enteral nutrition feeding route.	1.982	0.940	0.912
Factor 5	B5. During enteral nutrition implementation, I will strictly adhere to hand hygiene.	1.495	0.686	0.914
	B7. Unless medically contraindicated, I will elevate the head of the bed by 30° to 45° for patients receiving enteral nutrition support.	1.361	0.648	0.915
	B8. During enteral nutrition feeding, I will monitor symptoms of feeding intolerance in patients (such as nausea, vomiting, reflux/aspiration, abdominal distension), and promptly report to the physician.	1.403	0.627	0.914
	B10. I will cease enteral nutrition when patients undergo emergency airway establishment/replacement procedures, such as endotracheal intubation or tracheostomy.	1.406	0.739	0.915
	B11. I will stop enteral nutrition when patients undergo bedside X‐ray examinations or endotracheal fibroscopy.	1.448	0.771	0.915
	B12. In cases where a patient's condition deteriorates requiring immediate surgery or is anticipated to undergo general anaesthesia surgery within 4–8 h, I will halt enteral nutrition.	1.306	0.626	0.914
	B18. In critically ill patients with upper gastrointestinal bleeding or intestinal ischemia, I will discontinue enteral nutrition.	1.369	0.693	0.914
Factor 6	B14. For patients with feeding intolerance, I will investigate and analyse the causes and consult with the physician to jointly decide whether to discontinue enteral nutrition.	1.639	0.807	0.911
	B16. I will halt enteral nutrition for patients in shock with non‐correctable conditions, requiring the gradual increase in vasopressor doses to maintain hemodynamic stability (MAP <50 mmHg).	2.031	1.242	0.914
	B17. For patients experiencing uncontrollable life‐threatening hypoxemia, hypercapnia, or acidosis, I will discontinue enteral nutrition.	1.822	1.045	0.914
	B19. For patients with increased bladder pressure (IAP >20 mmHg), I will discontinue enteral nutrition.	1.785	0.978	0.916
	B21. During enteral nutrition support, if gastric residual volume is monitored twice and exceeds 250 mL, I will remind the physician about the potential use of prokinetic agents.	1.908	1.047	0.912
	B22. For patients intolerant to gastric feeding and ineffective use of prokinetic agents, or those deemed to have a high risk of aspiration, I will communicate with the physician to establish post‐pyloric feeding routes (inserting a “bullet head” nasointestinal tube for the patient).	1.678	0.853	0.914

### 3.5 Validity analysis results

#### 3.5.1 Construct validity

**FIGURE 1:**
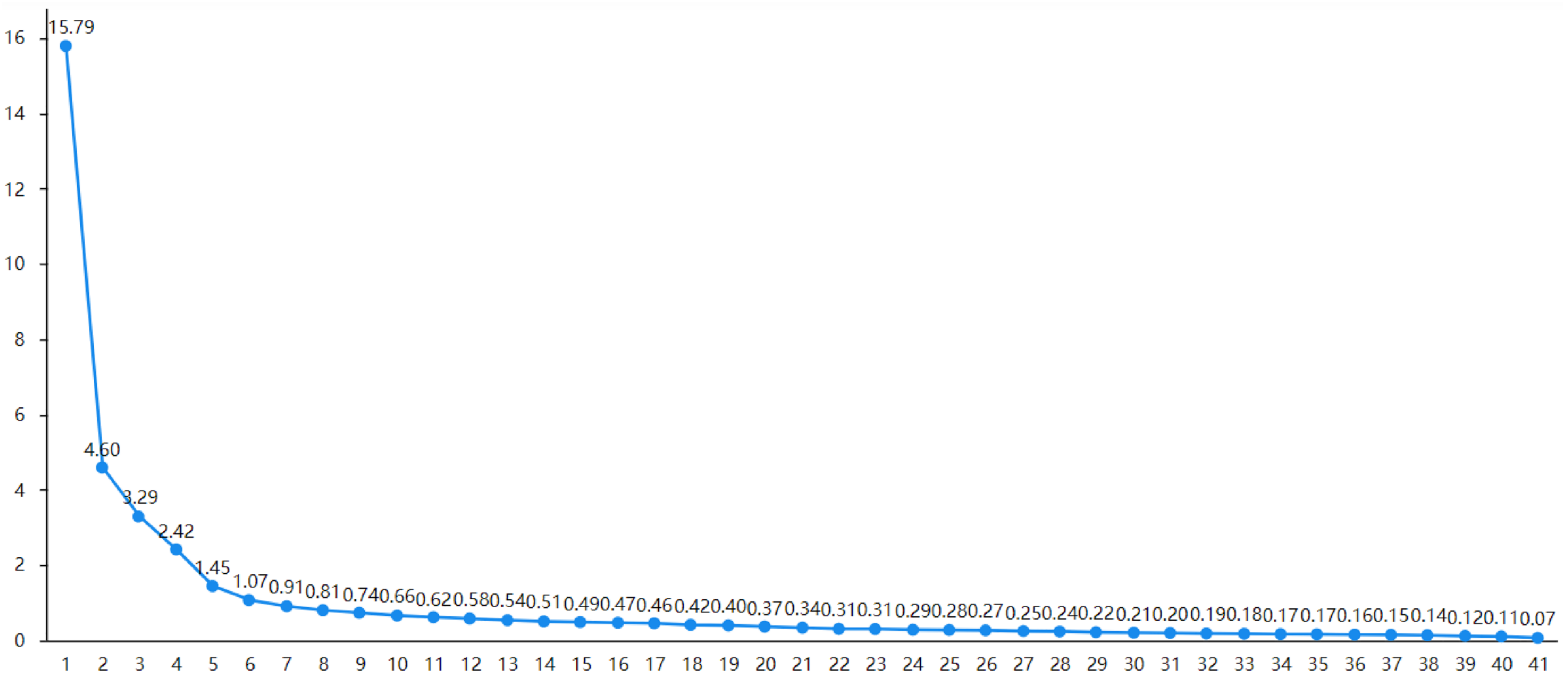
Scale entry gravel chart.

The Kaiser‐Meyer‐Olkin (KMO) values for the knowledge, attitude and practice dimensions of the scale were 0.938, 0.934 and 0.935, respectively. Bartlett's test of sphericity indicated significant differences (*p* < .001), confirming that the data were suitable for factor analysis. Six primary factors were extracted from the scale, in alignment with the scree plot (Figure 1). These factors were categorized as follows: two factors within the knowledge dimension (concepts and consequences of enteral nutritional interruption, and causes and influencing factors of interruption); one factor within the attitude dimension (perceptions/attitudes toward nutritional interruption); and three factors within the practice dimension (Learning and Communication, Medical Operation Behaviour, and Patient Disease Status).

##### Factor analysis results of the knowledge dimension

In the knowledge dimension, 16 entries were initially analysed, and 2 factors were extracted, each containing at least 3 entries. However, 2 entries (K5 and K7) showed dual loadings (both with loading values >0.5 on 2 factors and a difference of <0.2), leading to their deletion. After two rounds of exploratory analysis, 14 entries were retained in two distinct factors. Post‐rotation, these factors explained a cumulative variance of 70.341%, as detailed in Table 1.

##### Factor analysis results of the attitude dimension

For the attitude dimension, all 10 entries were subjected to factor analysis, leading to the extraction of one factor. Each entry had a factor loading greater than 0.6 and a commonality value exceeding 0.4. Post‐rotation, the factor explained 70.437% of the cumulative variance, as detailed in Table 1.

##### Factor analysis results of the practice dimension

In the practice dimension, 22 entries were initially analysed, resulting in the extraction of 3 factors. However, entry B6 showed dual loading, and entry B23 had a factor loading below 0.5, leading to their removal. After reanalysis with 20 entries, entry B9 showed a loading below 0.5, and entries B13 and B20 had commonality values below 0.4, resulting in their deletion. After three rounds of analysis, 17 entries across 3 distinct factors remained, with a cumulative variance of 66.550% after rotation, as outlined in Table 1.

#### 3.5.2 Content validity

The content validity index (CVI) was calculated based on expert ratings of each item's importance. The overall CVI for the scale was 0.975, while individual item CVIs ranged from 0.800 to 1.000, demonstrating strong content validity and comprehensiveness in assessing the scale's targeted domains.

### 3.6 Reliability analysis results

The Inter‐Factor Correlation Matrix is given in Table 3, which shows the correlations between the latent factors extracted from the scale. It can be seen that the correlations mostly range from 0.167 to 0.635, suggesting a moderate to strong relationship between some factors.

**TABLE 3:** Inter‐factor correlation matrix.

Factors	Factor 1	Factor 2	Factor 3	Factor 4	Factor 5	Factor 6
Factor 1	1.000	0.554	0.270	0.516	0.167	0.349
Factor 2		1.000	0.478	0.442	0.490	0.371
Factor 3			1.000	0.401	0.635	0.437
Factor 4				1.000	.408	0.544
Factor 5					1.000	0.622
Factor 6						1.000

Item total statistics are presented in Table 4, the Cronbach's α coefficient values are consistent (range: 0.911–0.954), indicating that no single item drastically affects the overall scale reliability.

**TABLE 4:** Item total statistics.

Dimensions	Item	Scale mean if item deleted	Scale variance if item deleted	Corrected item‐total correlation	Squared multiple correlation	Cronbach's alpha if item deleted
Knowledge	K1. An interruption in enteral nutrition feeding occurs when the infusion lasts for 1 h or more without continuous feeding.	26.675	76.477	0.478	0.548	0.936
	K2. Interruption of enteral nutrition feeding is defined as failure to receive the expected nutrients within 30 min, particularly in intermittent infusion occurring three times a day for 30 min each.	26.445	76.694	0.422	0.578	0.939
	K3. Post‐enternal nutrition interruptions may impact a patient's energy intake, increasing the risk of nutritional deficiencies.	27.191	73.116	0.692	0.610	0.929
	K4. Interruptions in enteral nutrition feeding correlate positively with patient severity, hospitalization costs and targeted caloric intake.	27.162	73.081	0.712	0.664	0.929
	K6. Factors such as hemodynamic instability, high intra‐abdominal pressure and digestion‐related complications (intestinal obstruction, anastomotic leakage, celiac disease) significantly contribute to enteral nutrition feeding interruptions.	27.453	73.010	0.779	0.667	0.927
	K8. Medical and nursing procedures like general anaesthesia, radiology, endotracheal fibre, airway establishment/replacement, position changes and suctioning often cause disruptions in enteral nutritional feeding.	27.466	73.394	0.787	0.709	0.926
	K9. Challenges such as tube placement difficulty, occlusion, displacement and dislodgement of nutritional infusion tubes are common causes of feeding interruptions in critically ill patients.	27.461	73.488	0.784	0.703	0.927
	K10. Early initiation of enteral nutrition within 24–48 h of admission is recommended for critically ill patients with well‐functioning gastrointestinal tracts and stable hemodynamics.	27.804	74.263	0.738	0.700	0.928
	K11. Monitoring the gastric remnant is crucial before each feeding for patients receiving enteral nutrition through fractionated push and intermittent gravity drip. For continuous nutrition pump infusion, monitoring should occur at least every 4 h.	27.673	74.415	0.715	0.696	0.929
	K12. Sedative and analgesic medications may delay gastric emptying. Regular assessment of pain levels and sedation depth is essential, minimizing drug use as per the treatment plan and patient's preferences.	27.573	73.510	0.787	0.738	0.926
	K13. Pro‐gastrointestinal dynamic drugs may alleviate gastrointestinal intolerance symptoms in critically ill patients.	27.605	73.494	0.804	0.755	0.926
	K14. For critically ill patients with elevated intra‐abdominal pressure (IAP >12 mmHg), routine monitoring of intra‐abdominal pressure is necessary. Adjusting the infusion rate and volume of enteral nutrition based on pressure levels is recommended.	27.343	73.239	0.729	0.582	0.928
	K15. Keeping the head of the bed elevated between 30° to 45° during enteral nutrition in critically ill patients is advisable unless contraindicated.	27.997	77.136	0.608	0.718	0.932
	K16. Resuming enteral nutritional feeding promptly after completing medical procedures, consultations and other related examinations is recommended.	27.890	76.345	0.685	0.751	0.930
Attitude	A1. It is crucial for ICU medical staff to possess knowledge about enteral nutrition feeding interruptions.	11.516	14.418	0.751	0.656	0.943
	A2. I believe that the hospital (or department) should conduct formal training on knowledge of enteral nutrition tolerance.	11.526	14.549	0.771	0.731	0.942
	A3. Enhanced knowledge regarding enteral nutrition feeding interruptions significantly aids in my clinical work.	11.505	14.529	0.789	0.701	0.941
	A4. Conducting nutritional assessments for all admitted ICU patients is regarded as an important practice.	11.542	14.517	0.801	0.688	0.941
	A5. Health care involvement is necessary for assessing the nutritional status of ICU patients.	11.615	14.857	0.844	0.800	0.940
	A6. The careful selection of appropriate enteral nutrition preparations/formulas plays a pivotal role in preventing interruptions in enteral nutritional feeding.	11.571	14.540	0.874	0.869	0.938
	A7. The proper establishment and selection of the infusion route for enteral nutrition are deemed important.	11.573	14.697	0.865	0.838	0.939
	A8. The management of enteral nutrition positioning holds significant importance.	11.545	14.574	0.806	0.743	0.941
	A9. Developing a standardized program for enteral nutrition management to prevent and address feeding interruptions is important	11.558	14.631	0.844	0.776	0.939
	A10. I believe that prioritizing the prevention of interruptions in enteral nutritional feeding outweighs treatment.	11.401	14.598	0.577	0.407	0.954
Practice	B1. I will proactively study relevant knowledge about interruptions in enteral nutrition feeding.	26.767	82.337	0.556	0.609	0.916
	B2. I will actively communicate with patients or their families about the importance of enteral nutrition, informing them about the risks of interruptions in enteral nutrition feeding.	26.741	81.568	0.557	0.653	0.916
	B3. Upon admitting ICU patients, I will promptly assess the patient's nutritional status and communicate with the attending physician.	27.076	81.803	0.629	0.670	0.914
	B4. Before implementing enteral nutrition, I will communicate with the doctor to select and establish the correct enteral nutrition feeding route.	27.110	80.959	0.670	0.708	0.912
	B5. During enteral nutrition implementation, I will strictly adhere to hand hygiene.	27.597	84.635	0.639	0.554	0.914
	B7. Unless medically contraindicated, I will elevate the head of the bed by 30° to 45° for patients receiving enteral nutrition support.	27.730	85.552	0.601	0.631	0.915
	B8. During enteral nutrition feeding, I will monitor symptoms of feeding intolerance in patients (such as nausea, vomiting, reflux/aspiration, abdominal distension) and promptly report to the physician.	27.688	85.018	0.672	0.662	0.914
	B10. I will cease enteral nutrition when patients undergo emergency airway establishment/replacement procedures, such as endotracheal intubation or tracheostomy.	27.686	84.846	0.572	0.526	0.915
	B11. I will stop enteral nutrition when patients undergo bedside X‐ray examinations or endotracheal fibroscopy.	27.644	84.356	0.581	0.559	0.915
	B12. In cases where a patient's condition deteriorates requiring immediate surgery or is anticipated to undergo general anaesthesia surgery within 4–8 h, I will halt enteral nutrition.	27.785	85.581	0.623	0.610	0.914
	B18. In critically ill patients with upper gastrointestinal bleeding or intestinal ischemia, I will discontinue enteral nutrition.	27.723	84.836	0.616	0.535	0.914
	B14. For patients with feeding intolerance, I will investigate and analyse the causes and consult with the physician to jointly decide whether to discontinue enteral nutrition.	27.453	82.070	0.715	0.534	0.911
	B16. I will halt enteral nutrition for patients in shock with non‐correctable conditions, requiring a gradual increase in vasopressor doses to maintain hemodynamic stability (MAP <50 mmHg).	27.270	80.523	0.617	0.632	0.914
	B17. For patients experiencing uncontrollable life‐threatening hypoxemia, hypercapnia, or acidosis, I will discontinue enteral nutrition.	27.306	81.573	0.603	0.602	0.914
	B19. For patients with increased bladder pressure (IAP >20 mmHg), I will discontinue enteral nutrition.	27.183	81.289	0.572	0.437	0.916
	B21. During enteral nutrition support, if gastric residual volume is monitored twice and exceeds 250 mL, I will remind the physician about the potential use of prokinetic agents.	27.414	82.128	0.668	0.523	0.912
	B22. For patients intolerant to gastric feeding and ineffective use of prokinetic agents, or those deemed to have a high risk of aspiration, I will communicate with the physician to establish post‐pyloric feeding routes (inserting a “bullet head” nasointestinal tube for the patient).	27.293	81.237	0.612	0.484	0.914

In Table 5, the mean score of the scale was 174.573 ± 19.424, the mean score in the knowledge dimension was 54.482 ± 9.266, the mean score in the attitude dimension was 47.183 ± 4.232, and the mean score in the practice dimension was 72.908 ± 9.653. The overall internal consistency of the scale, as indicated by a Cronbach's α coefficient, was 0.953. For subscale, Cronbach's α values ranged from 0.919 to 0.947. The scale also showed good stability with a test–retest reliability coefficient of 0.977, and test–retest reliability for the subscale ranged from 0.488 to 0.836. These metrics suggest the scale exhibits excellent internal consistency and satisfactory test–retest reliability.

**TABLE 5:** Mean score, internal consistency and test–retest reliability results for subscale.

Dimensions	Mean scores	MD	Cronbach's α	Test–Retest reliability
Knowledge	54.482	9.266	0.934	0.686
Attitude	47.183	4.232	0.947	0.488
Practice	72.908	9.653	0.919	0.836
Total Score	174.573	19.424	0.953	0.977

### 3.7 Final version of the scale

The final ICU Enteral Feeding Interruption knowledge, attitudes and practices (KAP) scale, after validity and reliability assessments, comprises 41 items across three dimensions, as shown in Appendix 1. The knowledge dimension includes 14 items under two factors: concepts and consequences of enteral feeding interruptions and factors influencing interruptions. Each item is rated on a 5‐point Likert scale (1 = “completely unaware,” 5 = “completely aware”), with a total possible score of 70. Higher scores indicate greater knowledge among ICU health care professionals. The attitude dimension consists of 10 items assessing perspectives on enteral nutrition interruptions, rated on a 5‐point Likert scale (1 = “completely disagree,” 5 = “completely agree”), with a total score of 50. Higher scores reflect a stronger recognition of the importance of managing feeding interruptions. The practice dimension includes 17 items covering learning and communication, medical procedures and patient condition. Items are rated from 1 (“never”) to 5 (“always”), with a total score of 85. Higher scores signify better adherence to enteral feeding interruption practices.

## 4 DISCUSSION

Presently, there is a multitude of tools worldwide for evaluating the nutritional status of critically ill patients, including the Nutrition Risk Screening 2002 (NRS2002),^24^ Nutrition Risk in the Critically Ill (NUTRIC)^25^ and Modified Nutrition Risk in the Critically Ill (mNUTRIC).^26^ These instruments serve as decision‐making aids for clinical nutritional interventions. However, the efficacy and accuracy of clinical decisions are shaped by medical cognition levels, divergent perspectives and operational behaviours in medical and nursing practices.^27^, ^28^, ^29^

Understanding health care professionals' knowledge, attitudes and practices is essential for developing targeted training programs. Currently, no dedicated tool exists for assessing ICU professionals' perspectives on enteral feeding interruptions. Developing a tailored assessment tool will enable the investigation of their knowledge, attitudes and practices, providing insights for management to formulate focused quality improvement strategies.

This study employed the knowledge, attitude and practice (KAP) framework, integrating extensive literature reviews, interviews with clinical staff and expert consultations to construct the scale. A meticulous process refined the item pool and initial draft, clarifying variables within the conceptual framework. For instance, the definition of enteral feeding interruption was elaborated to include support methods, frequency and duration, emphasizing the need for a comprehensive assessment of interruption factors.

To refine the scale's content and ensure its relevance, in‐depth interviews were conducted with clinicians and nursing experts specializing in enteral nutrition. Their feedback enhanced the precision of language, clarity and logical sequencing of items. Two rounds of expert consultations followed, utilizing the Delphi method with 20 experts from 15 tertiary hospitals across eight provinces in China, alongside methodologists. The expert authority coefficients reached 0.92 and 0.93. Item revisions and sequence adjustments were based on these consultations, with a Kendall's coefficient of 0.613 in the second round, reflecting strong consensus.

The scale underwent discriminant analysis, total‐item correlation and exploratory factor analysis (EFA), supported by scree plots. All metrics met recommended thresholds,^30^ affirming the scale's robustness. According to the methodology of scale development, it is mentioned that the time for retest reliability is generally between 2 and 4 weeks.^16^ Based on discussions within the research team, to avoid overestimating the retest ability, we chose a 30‐day interval for assessing test–retest reliability. This rigorous approach strengthens the tool's validity and reliability, making it an effective instrument for evaluating ICU health care professionals' knowledge, attitudes and practices (KAP) regarding enteral feeding interruptions.

This KAP assessment tool for ICU health care professionals has significant international relevance, especially for critical care nurses in diverse settings. While the scale was developed based on clinical and cultural contexts in China, its theoretical foundation—the knowledge, attitude and practice (KAP) framework—is universally applicable. The challenges of enteral feeding interruptions, including insufficient knowledge, suboptimal attitudes and inconsistent practices, are global issues in critical care. Studies from various countries have reported similar barriers to optimal enteral nutrition delivery, such as inconsistent protocols, lack of awareness and insufficient training.^31^, ^32^ Key features of this tool, such as its comprehensive consideration of interruption frequency, duration and underlying causes, make it adaptable to different health care systems. With minor contextual modifications, the scale can serve as a benchmark for assessing critical care nurses' competencies in managing enteral feeding interruptions worldwide.

Enteral feeding interruptions often result from health care professionals' insufficient knowledge and attention. Evaluating current knowledge and offering targeted training can update outdated perspectives and emphasize the importance of managing these interruptions. The scale serves as a practical tool, encouraging reflection and review of clinical practices, and fostering the integration of knowledge, attitudes and behaviours. Regular use of this scale can improve ICU staff's ability to manage enteral nutrition, enhancing patient outcomes and reducing complications caused by interruptions.

### 4.1 Limitations

This study has limitations. Convenience sampling may limit the representativeness of the findings. The lack of on‐site supervision could affect online survey quality. Participants were all from a single hospital in Wuhan, and regional differences in culture, health care policies and ICU practices suggest the need for further validation with a larger, more diverse sample for broader applicability. Lastly, as this is a cross‐sectional study, the responsiveness cannot be tested. Because of the lack of a recognized gold standard tool for KAP in this specific area, criterion validity has not yet been assessed. In future studies, we plan to establish the judgment criteria for the scale in conjunction with professional practice after completing external validation.

### 4.2 Implications and recommendations for practice

Critical care nurses play a pivotal role in ensuring the continuity of enteral nutrition, given their constant bedside presence and responsibility for monitoring and managing feeding protocols. The EFI Management KAP Scale is a valuable addition to the global effort to enhance enteral nutrition practices in ICUs. It provides a structured framework to identify knowledge gaps and areas for targeted professional development, enhancing nurses' understanding of the importance of uninterrupted EN. By standardizing clinical protocols and fostering adherence to evidence‐based practices, the tool supports nurses in effectively managing EN interruptions, thereby minimizing variability in care quality. Additionally, it promotes reflective practice, encouraging critical care nurses to continuously evaluate and improve their clinical approaches. The tool also facilitates multidisciplinary collaboration by offering insights into team‐wide practices, enabling cohesive efforts among nurses, physicians and dietitians to optimize EN delivery. Ultimately, its routine application can empower critical care nurses to play a proactive role in improving patient outcomes, such as maintaining adequate nutritional intake, reducing complications and promoting recovery in critically ill patients.

## 5 CONCLUSION

This EFI Management KAP Scale is a valuable addition to the global effort to enhance enteral nutrition practices in ICUs. Focusing on critical care nurses' knowledge, attitudes and practices, it provides actionable insights for education and quality improvement. Its potential for international adaptation underscores its broad utility in advancing critical care nursing standards. Integrating this tool into routine clinical practice can not only standardize care but also empower nurses to deliver more effective and patient‐centred nutritional support, ultimately improving outcomes for critically ill patients.

## FUNDING INFORMATION

This research was supported by The Healthcare Quality Management Research Project of the National Institute of Hospital Administration, National Health Commission (YLZLXZ24G073); Wuhan Union Hospital Pharmaceutical‐Technical‐Nursing Fund project (Project No. 02.03.2021‐35).

## CONFLICT OF INTEREST STATEMENT

The authors declare that the research was conducted in the absence of any commercial or financial relationships that could be construed as a potential conflict of interest.

## Data Availability

The data that support the findings of this study are available from the corresponding author upon reasonable request.

## APPENDIX 1 KAP SCALE OF ENTERAL NUTRITION INTERRUPTION

**Table:**

Items	Scores				
	5	4	3	2	1
Knowledge dimension	Completely familiar	Highly familiar	Moderately familiar	Uncertain	Completely unfamiliar
1. Enteral nutrition feeding is considered interrupted when the infusion lasts for 1 h or more without continuous feeding.					
2. Interruption of enteral nutrition feeding is defined as the failure to receive the expected nutrients within 30 min, particularly in intermittent infusion occurring three times a day for 30 min each.					
3. Enteral nutrition interruptions may impact a patient's energy intake, increasing the risk of nutritional deficiencies.					
4. Interruptions in enteral nutrition feeding correlate positively with patient severity, hospitalization costs and targeted caloric intake.					
5. Factors such as hemodynamic instability, high intra‐abdominal pressure and digestive‐related complications (intestinal obstruction, anastomotic leakage, celiac disease) significantly contribute to interruptions in enteral nutrition feeding.					
6. Medical and nursing procedures such as general anaesthesia, radiology, endotracheal fiberscopy, airway establishment/replacement, position changes, and suctioning often result in disruptions to enteral nutritional feeding.					
7. Challenges like tube placement difficulty, occlusion, displacement, and dislodgement of nutritional infusion tubes commonly lead to feeding interruptions in critically ill patients.					
8. Early initiation of enteral nutrition within 24–48 h of admission is recommended for critically ill patients with well‐functioning gastrointestinal tracts and stable hemodynamics.					
9. Monitoring the gastric remnant before each feeding is crucial for patients receiving enteral nutrition through fractionated push and intermittent gravity drip. For continuous nutrition pump infusion, monitoring should occur at least every 4 h.					
10. Sedative and analgesic medications may delay gastric emptying. Regular assessment of pain levels and sedation depth is essential, minimizing drug use according to the treatment plan and patient's preferences.					
11. Pro‐gastrointestinal dynamic drugs may alleviate gastrointestinal intolerance symptoms in critically ill patients.					
12. For critically ill patients with elevated intra‐abdominal pressure (IAP >12 mmHg), routine monitoring of intra‐abdominal pressure is necessary. Adjusting the infusion rate and volume of enteral nutrition based on pressure levels is recommended.					
13. Keeping the head of the bed elevated between 30° to 45° during enteral nutrition in critically ill patients is advisable unless contraindicated.					
14. Resuming enteral nutritional feeding promptly after completing medical procedures, consultations, and other related examinations is recommended.					
Attitude dimension	Strongly agree	moderate agree	slightly agree	Disagree	Strongly disagree
1. It is crucial for ICU medical staff to possess knowledge about enteral nutrition feeding interruptions.					
2. I believe that the hospital (or department) should conduct formal training on knowledge of enteral nutrition tolerance.					
3. Enhanced knowledge regarding enteral nutrition feeding interruptions significantly aids in my clinical work.					
4. Conducting nutritional assessments for all admitted ICU patients is regarded as an important practice.					
5. Health care involvement is necessary for assessing the nutritional status of ICU patients.					
6. The careful selection of appropriate enteral nutrition preparations/formulas plays a pivotal role in preventing interruptions in enteral nutritional feeding.					
7. The proper establishment and selection of the infusion route for enteral nutrition are deemed important.					
8. The management of enteral nutrition positioning holds significant importance.					
9. Developing a standardized program for enteral nutrition management to prevent and address feeding interruptions is important					
10. I believe that prioritizing the prevention of interruptions in enteral nutritional feeding outweighs treatment.					
Practice dimension	Always	Often	Sometimes	Occasionally	Never
1. I will diligently study relevant information concerning interruptions in enteral nutrition feeding.					
2. I will actively engage with patients or their families, emphasizing the significance of enteral nutrition and the associated risks of interruptions.					
3. Upon admitting ICU patients, I will promptly evaluate their nutritional status and collaborate with the attending physician.					
4. Before initiating enteral nutrition, I will consult with the physician to determine and establish the appropriate feeding route.					
5. Throughout the implementation of enteral nutrition, I will rigorously adhere to hand hygiene practices.					
6. Unless medically contraindicated, I will incline the head of the bed by 30° to 45° for patients receiving enteral nutrition support.					
7. While administering enteral nutrition, I will vigilantly monitor patients for signs of feeding intolerance (such as nausea, vomiting, reflux/aspiration, abdominal distension), promptly notifying the physician of any concerns.					
8. I will suspend enteral nutrition during emergency airway establishment/replacement procedures, such as endotracheal intubation or tracheostomy.					
9. I will suspend enteral nutrition during bedside X‐ray examinations or endotracheal fibroscopy procedures.					
10. If a patient's condition deteriorates, requiring immediate surgery or anticipated general anaesthesia within 4–8 h, I will discontinue enteral nutrition.					
11. In cases of feeding intolerance, I will investigate the underlying causes, consult with the physician, and collectively decide whether to cease enteral nutrition.					
12. Enteral nutrition will be halted for patients in shock with irreversible conditions, necessitating a gradual increase in vasopressor doses to maintain hemodynamic stability (mean arterial pressure <50 mmHg).					
13. Enteral nutrition will be discontinued for patients experiencing uncontrollable life‐threatening hypoxemia, hypercapnia, or acidosis.					
14. In critically ill patients with upper gastrointestinal bleeding or intestinal ischemia, enteral nutrition will be terminated.					
15. For patients with elevated bladder pressure (intra‐abdominal pressure >20 mmHg), enteral nutrition will be ceased.					
16. If gastric residual volume is monitored twice and exceeds 250 mL during enteral nutrition support, I will alert the physician about the potential use of prokinetic agents.					
17. For patients intolerant to gastric feeding despite prokinetic agent use, or those at high risk of aspiration, I will collaborate with the physician to establish post‐pyloric feeding routes (inserting a “bullet head” nasointestinal tube for the patient).					

## References

[1] Baiu I , Spain DA . Enteral nutrition. JAMA. 2019;321:2040. doi:10.1001/jama.2019.4407 31135851

[2] Koretz RL . Early enteral nutrition in the ICU. Intensive Care Med. 2010;36:1087‐1088; author reply 1089‐90. doi:10.1007/s00134-010-1788-6 20213071 PMC2864896

[3] McClave SA , DiBaise JK , Mullin GE , Martindale RG . ACG clinical guideline: nutrition therapy in the adult hospitalized patient. Am J Gastroenterol. 2016a;111:315‐334; quiz 335. doi:10.1038/ajg.2016.28 26952578

[4] Viladomiu M , Hontecillas R , Yuan L , Lu P , Bassaganya‐Riera J . Nutritional protective mechanisms against gut inflammation. J Nutr Biochem. 2013;24:929‐939. doi:10.1016/j.jnutbio.2013.01.006 23541470 PMC3730123

[5] McClave SA , Taylor BE , Martindale RG , et al. Guidelines for the provision and assessment of nutrition support therapy in the adult critically ill patient: Society of Critical Care Medicine (SCCM) and American Society for Parenteral and Enteral Nutrition (a.S.P.E.N.). JPEN J Parenter Enteral Nutr. 2016b;40:159‐211. doi:10.1177/0148607115621863 26773077

[6] Singer P , Blaser AR , Berger MM , et al. ESPEN guideline on clinical nutrition in the intensive care unit. Clin Nutr. 2019;38:48‐79. doi:10.1016/j.clnu.2018.08.037. 30348463

[7] Lee ZY , Ibrahim NA , Mohd‐Yusof BN . Prevalence and duration of reasons for enteral nutrition feeding interruption in a tertiary intensive care unit. Nutrition. 2018;53:26‐33. doi:10.1016/j.nut.2017.11.014 29627715

[8] Salciute‐Simene E , Stasiunaitis R , Ambrasas E , et al. Impact of enteral nutrition interruptions on underfeeding in intensive care unit. Clin Nutr. 2021;40:1310‐1317. doi:10.1016/j.clnu.2020.08.014 32896448

[9] Boeykens K , Van Hecke A . Advanced practice nursing: nutrition nurse specialist role and function. Clin Nutr ESPEN. 2018;26:72‐76. doi:10.1016/j.clnesp.2018.04.011 29908686

[10] Malhi H , Dera M , Fletcher J . Exploring the role of the nutrition nurse specialist in an intestinal failure tertiary referral centre. Br J Nurs. 2022;31:S4‐S12. doi:10.12968/bjon.2022.31.7.S4 35404659

[11] Mogre V , Stevens F , Aryee PA , Amalba A , Scherpbier A . Future doctors' perspectives on health professionals' responsibility regarding nutrition care and why doctors should learn about nutrition: a qualitative study. Educ Health Abingdon. 2019;32:91‐94. doi:10.4103/efh.EfH_134_17 31745003

[12] Chakraborty S , Kopsco H , Evans C , Mateus‐Pinilla N , Smith RL . Assessing knowledge gaps and empowering extension workers in Illinois with information on ticks and tickborne diseases through KAP surveys. Heliyon. 2024;10:e25789. doi:10.1016/j.heliyon.2024.e25789 38352775 PMC10862665

[13] Wakui N , Kikuchi M , Ebizuka R , et al. Survey of Pharmacists' knowledge, attitudes, and practices (KAP) concerning COVID‐19 infection control after being involved in vaccine preparation: a cross‐sectional study. Int J Environ Res Public Health. 2022;19:9035. doi:10.3390/ijerph19159035 35897405 PMC9331880

[14] Yang H , Zhang H , Lu Y , Gu Y , Zhou J , Bai Y . A program to improve the knowledge, attitudes, and practices of needle stick and sharps injuries through bundled interventions among nurses: an KAP mode‐based approach to intervention. Psychol Health Med. 2022;27:999‐1010. doi:10.1080/13548506.2020.1830132 33048583

[15] Gorsuch RL . Factor Analysis: Classic Edition. 2nd ed. Routledge; 2014.

[16] Minglong W . Statistical Analysis of Scales: Operation and Application of SPSS. Chongqing University Press; 2010.

[17] Campbell S , Greenwood M , Prior S , et al. Purposive sampling: complex or simple? Research case examples. J Res Nurs. 2020;25:652‐661. doi:10.1177/1744987120927206 34394687 PMC7932468

[18] Crowe M , Inder M , Porter R . Conducting qualitative research in mental health: thematic and content analyses. Aust N Z J Psychiatry. 2015;49:616‐623. doi:10.1177/0004867415582053 25900973

[19] Boateng GO , Neilands TB , Frongillo EA , Melgar‐Quinonez HR , Young SL . Best practices for developing and validating scales for health, social, and behavioral research: a primer. Front Public Health. 2018;6:149. doi:10.3389/fpubh.2018.00149 29942800 PMC6004510

[20] Sun R , Jiang R , Huang M , Cai G . Consensus of early enteral nutrition clinical practice in critically ill patients. Zhonghua Wei Zhong Bing Ji Jiu Yi Xue. 2018;30:715‐721. doi:10.3760/cma.j.issn.2095-4352.2018.08.001 30220270

[21] Mi Y , Huang H , Shang Y , et al. Expert consensus on prevention and management of enteral nutrition therapy complications for critically ill patients in China (2021 edition). Zhonghua Wei Zhong Bing Ji Jiu Yi Xue. 2021;33:903‐917. doi:10.3760/cma.j.cn121430-20210310-00357 34590555

[22] Mccoach DB , Gable RK , Madura JP . Instrument Development in the Affective Domain: Defining, Measuring, and Scaling Affective Constructs. Springer; 2013.

[23] Sun D , Zheng R . Psychometric Theory. Kaiming Press; 2012.

[24] Coruja MK , Cobalchini Y , Wentzel C , Fink J . Nutrition risk screening in intensive care units: agreement between NUTRIC and NRS 2002 tools. Nutr Clin Pract. 2020;35:567‐571. doi:10.1002/ncp.10419 31602679

[25] Rosa M , Heyland DK , Fernandes D , Rabito EI , Oliveira ML , Marcadenti A . Translation and adaptation of the NUTRIC score to identify critically ill patients who benefit the most from nutrition therapy. Clin Nutr ESPEN. 2016;14:31‐36. doi:10.1016/j.clnesp.2016.04.030 28531396

[26] Mendes R , Policarpo S , Fortuna P , Alves M , Virella D , Heyland DK . Nutritional risk assessment and cultural validation of the modified NUTRIC score in critically ill patients‐a multicenter prospective cohort study. J Crit Care. 2017;37:45‐49. doi:10.1016/j.jcrc.2016.08.001 27621112

[27] Kane NB, Keene AR, Owen GS , et al. Applying decision‐making capacity criteria in practice: A content analysis of court judgments. PLoS One. 2021;16:e0246521. doi:10.1371/journal.pone.0246521 33544766 PMC7864395

[28] Hermann H , Trachsel M , Elger BS , Biller‐Andorno N . Emotion and value in the evaluation of medical decision‐making capacity: a narrative review of arguments. Front Psychol. 2016;7:765. doi:10.3389/fpsyg.2016.00765 27303329 PMC4880567

[29] Riiser A , Andersen V , Saeterbakken A , Ylvisaker E , Moe VF . Running performance and position is not related to decision‐making accuracy in referees. Sports Med Int Open. 2019;3:E66‐E71. doi:10.1055/a-0958-8608 31428673 PMC6697522

[30] Sun Z , Xu Y . Medical Statistics. 4th ed. People's Medical Publishing House; 2014.

[31] Heyland DK , Lemieux M , Shu L , Quisenberry K , Day AG . What is “best achievable” practice in implementing the enhanced protein‐energy provision via the enteral route feeding protocol in intensive care units in the United States? Results of a multicenter, quality improvement collaborative. JPEN J Parenter Enteral Nutr. 2018;42:308‐317. doi:10.1177/0148607116673301 27875285

[32] Preiser JC , Arabi YM , Berger MM , et al. A guide to enteral nutrition in intensive care units: 10 expert tips for the daily practice. Crit Care. 2021;25:424. doi:10.1186/s13054-021-03847-4 34906215 PMC8669237

